# Investigating the feasibility and effectiveness of a modular treatment program for children and adolescents with depression and interpersonal problems: study protocol of a quasi-experimental pilot feasibility trial (CBASP@YoungAge)

**DOI:** 10.1186/s40814-022-01091-3

**Published:** 2022-07-11

**Authors:** N. Dippel, T. In-Albon, S. Schneider, H. Christiansen, E.-L. Brakemeier

**Affiliations:** 1 Philipps-University Marburg, Marburg, Germany; 2 University of Koblenz-Landau, Landau, Germany; 3 Ruhr-University Bochum, Bochum, Germany; 4 University of Greifswald, Greifswald, Germany

**Keywords:** Depression, Psychotherapy, Children, Adolescents, Caregiver involvement, Modular treatment, CBASP

## Abstract

**Background:**

Depression is a serious disorder in childhood and adolescence. Affected children and adolescents show significant impairments in various aspects of life. Studies on the effectiveness or efficacy of psychotherapy in depressed children and adolescents are qualitatively very heterogeneous and reveal small effect sizes. There is thus a need to better tailor psychotherapy approaches to these age groups to improve outcomes like parent-child relationship, symptomatology, or quality of life.

To address this gap, we designed a modular, individualized treatment program for children and adolescents based on the Cognitive Behavioral Analysis System of Psychotherapy (CBASP) including caregiver involvement.

**Method:**

This quasi-experimental pilot feasibility trial is a phase 1 to phase 2 study investigating the feasibility and effectiveness of CBASP@YoungAge by including an intervention group (CBASP@YoungAge) and a treatment-as-usual control group. The treatment of depressive symptoms as well as interpersonal problems with primary caregivers are the main targets of CBASP@YoungAge. Personalization is ensured concerning the treatment course, caregivers’ involvement, and the patient’s age. The primary outcome relates to two areas: the feasibility of the CBASP@YoungAge treatment program in an outpatient context and a change in patients' depressive symptomatology from before to after treatment. We conduct a brief process evaluation after each session in the intervention group to closely monitor the treatment process and examine feasibility from the therapists' and patients' perspectives and mechanisms of symptom change. In addition, we consider interpersonal behavior between children and caregivers, parenting behavior, and monitor the global-health-index in children and parents as secondary outcomes. Pre-, post-, and follow-up data are evaluated.

**Discussion:**

This is the first study of a modular-based intervention program for children and adolescents with depression and a clear focus on the interpersonal problems between the depressed young patient and her/his caregiver. It will provide important knowledge on the feasibility and effectiveness of the program and potential benefits of including caregivers in psychotherapy. Based on this study’s results, we plan a multicenter, randomized, controlled trial whose long-term aim is to improve the psychotherapeutic care of young patients with depression while preventing persistent courses of depressive disorders.

**Trial registration:**

German Clinical Trials Register, DRKS (identifier DRKS00023281). Registered 17 November 2020–Retrospectively registered

**Supplementary Information:**

The online version contains supplementary material available at 10.1186/s40814-022-01091-3.

## Background

Depression in childhood and adolescence is widespread and debilitating at every stage of development [1–4]. Children and adolescents suffer from the same main depressive symptoms as adults, however, children and adolescents often perform worse at school, have social network difficulties, and somatoform symptoms [5, 6]. When depressive symptoms are not alleviated, the disorder tends to persist in adulthood [7, 8]. Early onset depression is associated with lower quality of life in childhood and adulthood, higher levels of comorbidities, and with other medical and mental disorders [9] as well as more suicide attempts [10–14]. Persistent negative family and social consequences in childhood or adolescence are also common [6].

These findings suggest that there is a strong need for effective treatment of depression in these age groups through early and evidence-based interventions [7, 15]. There is growing research on the psychotherapy of depression in children and adolescents, but recent studies have delivered heterogeneous results and only low to medium effect sizes [16–20]. Reviews focusing exclusively on the psychotherapy of depression in childhood and adolescence [16, 21] reported an overall effect size (g) of 0.36 at post-treatment and 0.21 at follow-up. A meta-analysis that focused on changes in child and adolescent depression-treatment effects over time revealed that mean effects drop significantly between post- and follow-up assessments [22].

This study thus aims to improve the treatment of depression in children and adolescents by focusing on how depression develops in children and adolescents as well as important causative and maintaining factors. As there is evidence of an association between the relationship quality of caregivers and their children in internalizing symptoms [23, 24], we assume that the interaction of children, adolescents and their caregivers is related to the development of depressive symptoms. Specifically, dysfunctional caregiver-child interaction behaviors appear to promote the development of depressive disorders in children and adolescents [25–29]. This association is, of course, also influenced by the attachment style. Especially anxious and avoidant attachment are relevant in this case, as those have been shown to be related to depressive symptoms [30, 31]. Accordingly, changes in interactional behavior or attachment of the parents should be drivers of change in child and adolescent depression symptomatology [32–34].

The Cognitive Behavioral Analysis System of Psychotherapy (CBASP) is a disorder-specific and integrative treatment approach that entails psychodynamic and cognitive-behavioral elements as well as theories from the interpersonal school [35]. CBASP was originally developed for adult patients who have developed chronic depression due to childhood maltreatment and negative relationship experiences, and as most of them have already undergone many different treatment attempts without success, they can be described as therapy-resistant [36]. CBASP addresses formative relationship experiences by first identifying significant others and the accompanying basic assumptions and thought patterns (causal conclusions, imprints). Actual relationship expectations or fears (transference hypotheses) are then documented to be processed and, at best, corrected through interpersonal and behavioral interventions [37, 38]. In this way, patients benefit from new positive relational experiences that enable a changed perception of the self and of others. Meanwhile, various studies on CBASP have been conducted in different settings and variations, and CBASP is considered an evidence-based psychotherapy for adults with depressive disorders [39–42]. However, positive studies have nevertheless revealed a high relapse and dropout rate, especially among young adults with depressive disorders [43]. As there are currently no studies on CBASP for the treatment of children and adolescents with depression, we report here on a pilot study for which we first adapted CBASP to target these age groups. Based on research findings on the involvement of caregivers in psychotherapy [44–46], and positively evaluated modular interventions [47, 48], we designed a modular, individualized treatment program for children and adolescents called CBASP@YoungAge. This study aims to implement CBASP@YoungAge in clinical practice and to investigate its feasibility and effectiveness compared to an active treatment as usual control group in a non-randomized design.

### Objective

The objective of this study is to assess the feasibility and potential effectiveness of the CBASP@YoungAge outpatient treatment program designed to treat children and adolescents with depression and interpersonal problems. The study aimed (1) to examine the acceptability and feasibility of the modular treatment program for therapists, patients, and caregivers. We assess whether the therapist employs the modular approach and if the patients and caregivers understand the material and treatment structure and participate in the treatment. Furthermore, the study aims to (2) investigate potential changes in the patients’ depressive symptoms and in the interpersonal behavior between patient and participating caregivers during the treatment process and compared to post-treatment. We expect (3) that this treatment will lead to a greater alleviation of depressive symptomatology, to improved interpersonal behavior of children and caregivers, and to stronger long-term alleviation of the patients' symptomatology than treatment-as-usual (TAU) group. In addition, we expect (4) to observe a significantly higher proportion of parent involvement in the intervention group than in the control group.

## Methods/Design

This pilot study is a non-randomized, quasi-experimental pilot feasibility trial comparing CBASP@YoungAge with a TAU control group (the actual gold standard in psychotherapy research [49]), to evaluate the feasibility and effectiveness of CBASP@YoungAge with respect to the depressive symptoms of children and adolescents and their interpersonal behavior with caregivers. This protocol is reported according to the SPIRIT 2013 [50] statement (see Additional file 1: completed SPIRIT checklist). For a brief overview, see the WHO Checklist (Additional file 2: WHO Trial Registration Data Set).

### Study setting

As CBASP@YoungAge is an outpatient treatment program, the intervention group is implemented at the Child and Adolescents Psychotherapy Outpatient Clinic Marburg (KJ-PAM, Philipps-University Marburg) while the TAU control groups are implemented at the Center for Child and Adolescent Psychotherapy (Outpatient Clinic, Ruhr-University Bochum) and the Landau Psychotherapy Outpatient Clinic for Children and Adolescents. All outpatient clinics are based in Germany and leading psychotherapy institutions invoice the German health insurance companies. The therapists are psychotherapy trainees.

### Participants and sample size

Eligible participants are aged between 10 and 21 years and meet the criteria for depressive disorders, based on DSM-5 [51]. Inclusion is based on a structural clinical interview (Kinder-DIPS, parent or child version [52]). A participating caregiver is mandatory in the CBASP@YoungAge group, whereas caregiver participation in the TAU groups is not requested beyond the legal requirements. As psychotherapy is conducted in German, children and participating parents need to be sufficiently fluent in German. Further, sufficient cognitive level of participating children/adolescents is required for the patients to be able to follow the cognitive interventions. Since intelligence tests are part of the standard diagnostics in the participating outpatient clinics, this criterion is operationalized by an IQ of ≥ 80.

We exclude children or adolescents in case of ongoing psychotherapy, acute suicidality, or a disorder expression requiring a different and non-depression focused treatment (e.g., traumatic disorder, schizophrenia, or bipolar disorders) or an inpatient stay. We do not exclude comorbid disorders or ongoing psychopharmacotherapy to enhance the external validity and generalizability of our findings. In case of outpatient housing, youth in the CBASP@YoungAge treatment group can nominate a different adult to participate in the sessions, as long as there is at least one significant person able and willing to join treatment sessions. If more than one person wants to participate, that is also possible.

Sample size calculations are guided by the considerations of Hertzog [53]. Thus, the planning of a larger study by this investigation was important, as well as the capacities of the centers involved and the sample size and drop out rate of other comparable studies [54, 55]. We aim to recruit 44 participants (22 per treatment arm). Assuming a 60% retention rate during the trial [56], this will lead 38 treatment completers with complete data.

### Recruitment and procedure

Since the design is non-randomized, patients are consecutively recruited in the participating outpatient clinics. After initial appointments, therapists in the outpatient clinics identify and approach eligible patients based on the inclusion and exclusion criteria. They participate either in the CBASP@YoungAge treatment arm or in the treatment-as-usual control group (TAU). If screening indicates that the patient is eligible for participation and the family consents, complete study information is given to all participants, and written informed consent is obtained from the primary caregiver and participating child or adolescent.

**Fig. 1:**
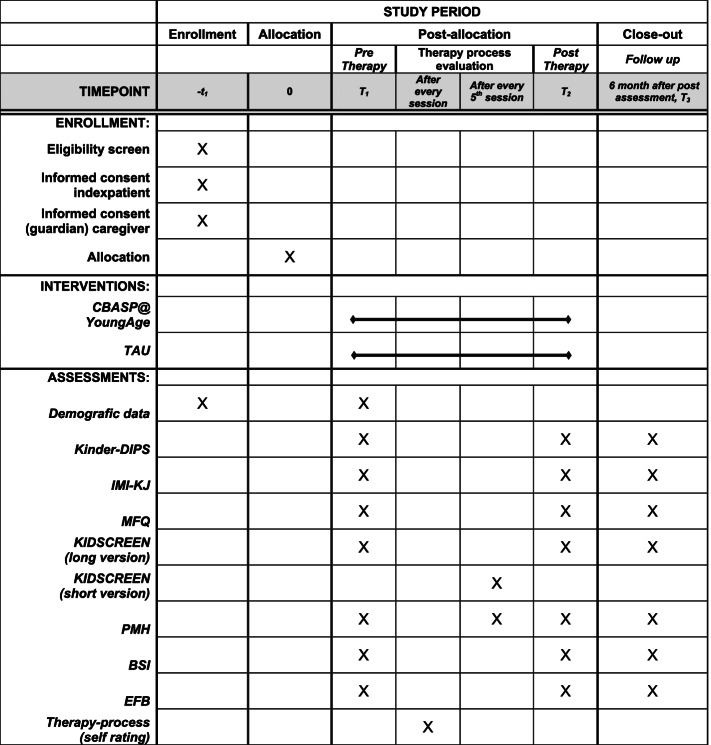
SPIRIT figure, schedule of enrolment, interventions, and assessments of the trial  [57]

**Fig. 2:**
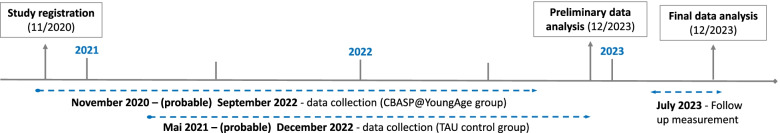
Timeline of the study

Assessments are at baseline, post-treatment, and 6 months follow-up. During the treatment phase, a quantitative and qualitative orientated therapy process evaluation by therapist, patient, and caregivers is planned for every session, as is a quantitative evaluation of the general health index by patient and caregivers every 5th session. For detailed information on the study procedure, see Fig. 1 and for an overview of the actual study process and timeline Fig. 2.

The psychotherapeutic process is evaluated during treatment by the study therapists. If difficulties arise during treatment, the study therapists contact the study team in the appropriate outpatient clinic. The intervention or follow-up is discontinued if the caregivers or patients withdraw consent or a different treatment becomes necessary. The therapy process is also discontinued if the trial has to be unexpectedly canceled, or if the study coordinator decides it is necessary to discontinue, for example in case of adverse events. The risk management information below provides further information. The date and reason for discontinuation or drop-outs after screening will be documented.

**Fig. 3:**
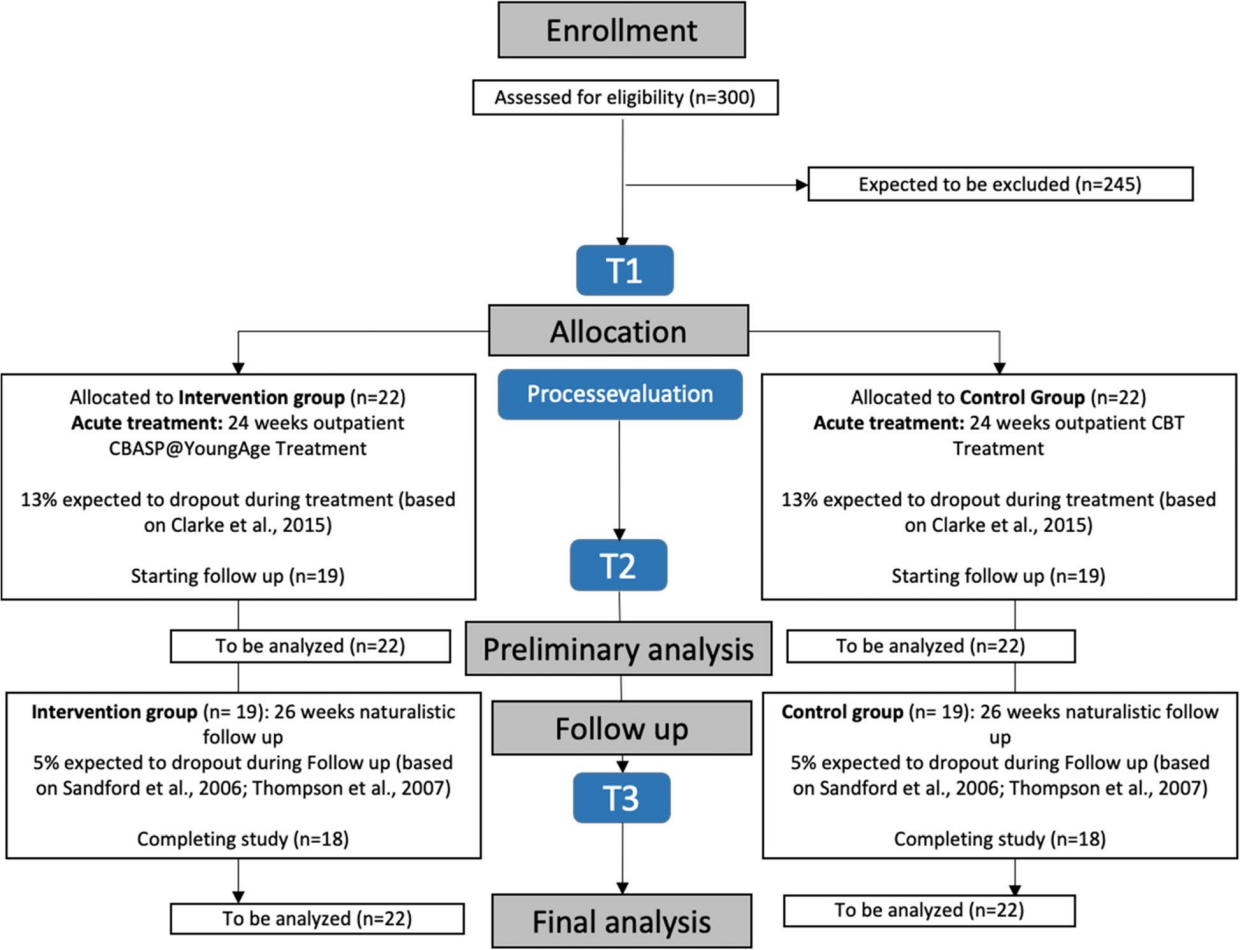
Flowchart of the Study design (based on CONSORT)

After treatment completion, trained therapists will carry out regular post-assessments who were not involved in the treatment. Participants have to wait for at least 6 weeks before booster sessions are allowed when needed. If follow-up treatment (e.g., for comorbidities) appears to be necessary during booster sessions, that can be implemented as part of regular outpatient treatment, and will be documented (see Fig. 3 for the study flow).

### Randomization, allocation concealment, and blinding

Assignment to the two study arms is non-randomized and the allocation is not blinded. The pre- and post-assessments will not be blinded, but rather done by trained psychotherapists who are independent and did not provide treatment in either of the study arms to the respective families.

### Trial interventions

#### CBASP@YoungAge intervention

**Fig. 4:**
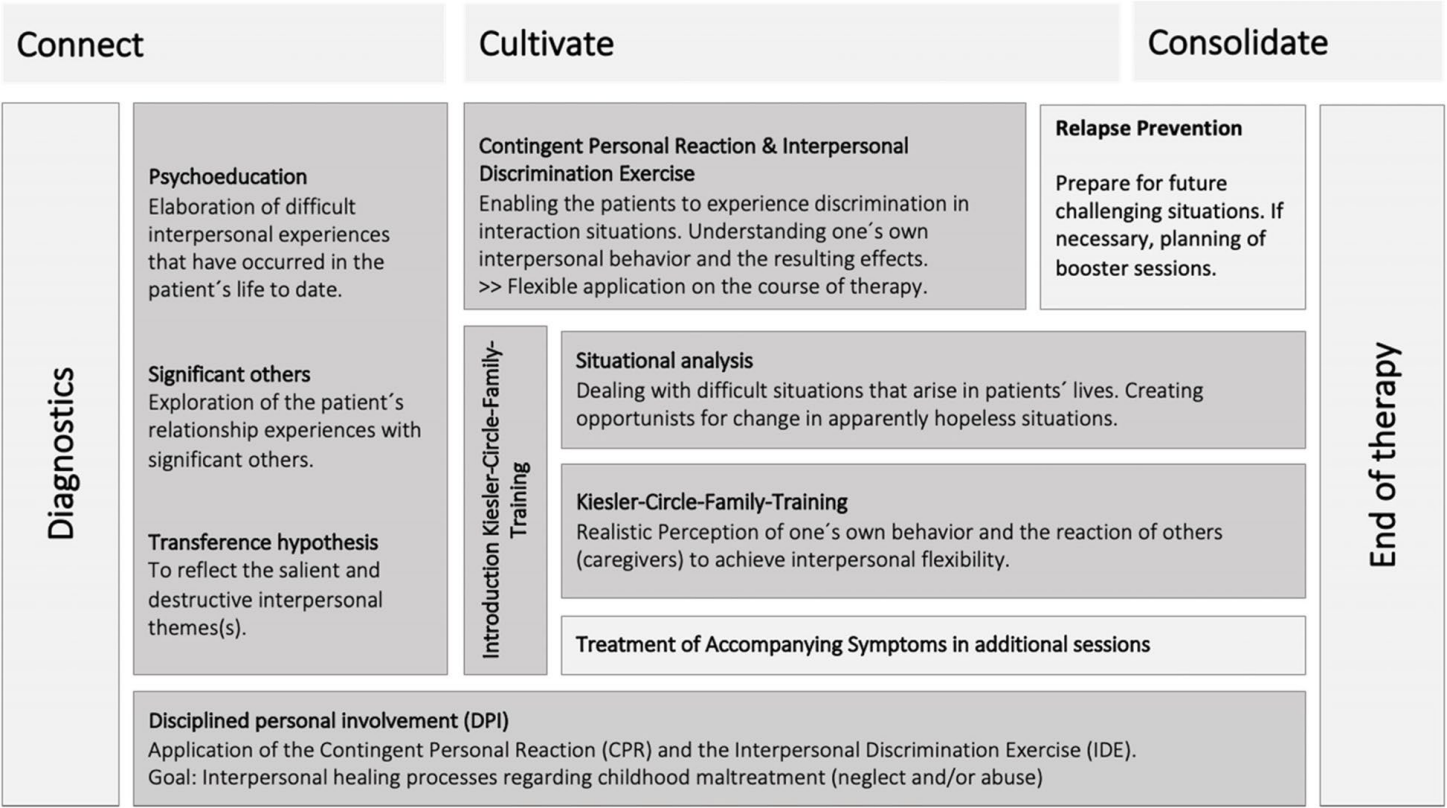
Procedure and structure of the treatment program (CBASP@YoungAge)

Based on CBASP [35], we designed the modular, individualized treatment program CBASP@YoungAge for children and adolescents that targets depressive symptoms while additionally addressing interactional difficulties with primary caregivers [58]. The treatment program is manualized and generally applicable in other outpatient psychotherapy contexts. CBASP@YoungAge differs from the traditional CBASP approach in several aspects. First, the active involvement of significant primary caregivers in therapy is mandatory. Second, in line with different previous studies, we modified CBASP more than originally intended [47, 59–62]. Third, the treatment approaches are age-adapted. For detailed information about the CBASP@YoungAge intervention, see the treatment manual (Dippel N, Christiansen H, Brakemeier E-L: CBASP@YoungAge – Ein Therapiemanual für das ambulante Setting, in preparation). A brief overview is given in Fig. 4 and in the text below.

CBASP@YoungAge is conceived for three different age groups (10–12, 13–16, 17–21 years) with depressive disorders and resulting interpersonal problems. It consists of nine different modules that combine aspects of cognitive behavioral therapy (CBT) and psychodynamic psychotherapy. Overall, the intervention consists of three treatment modalities. The first is the individual treatment format, where the therapists work with the child or adolescent on their specific issues. Additional individual sessions with the caregivers are also possible. Second, in the joint format the patient and related caregiver share one session where both work individually on the same topic with the therapist. Third, the joint format the patient and the caregiver participate in the therapy session together. In general, the choice of the appropriate format is left to the therapist, as individual structural conditions must also be considered. The treatment manual only makes recommendations.

This structure aims to provide the most individualized and personalized course of treatment possible while supporting the therapist in every variation. To personalize the treatment, therapists have various age-appropriate adaptation options in the material, as well as flexibility in handling the sequence and the modules’ duration.

The therapeutic relationship in CBASP and thus also CBASP@YoungAge is called “Disciplined Personal Involvement” (DPI) [38] and is characterized by an appreciative, open, and authentic basic attitude towards the patient. DPI is a special form of relationship building that includes the therapist’s personal inner world, thinking, emotions, and reactions to the patient’s behavior [58].

The connecting phase focuses on psychoeducation as well as elaborating on the involvement of significant others and the CBASP-specific transference hypothesis. The connecting phase follow a fixed order to establish the therapy basis. During this phase, an individual model of depression within the CBASP@YoungAge therapy-rationale is developed based on the “interpersonal wall” concept. This wall describes metaphorically the difficulty patients have in interacting appropriately with their social environment. We are assuming that, because of previous experiences, youths’ skills and abilities are impaired in relating to significant others or in interpreting the behavior of others accurately. Such (early) relationship difficulties are assumed to result in hopelessness, distrustfulness, helplessness and loneliness, and reduced contact with other people or activities. Overall, the connecting phase focuses on difficult past and present relationship experiences to establish the transference hypothesis as an overall imprint for daily interactions and the current psychotherapeutic work (e.g., “If I open up to someone and share my emotions, that person won't care and won't react, so I keep my feelings to myself and react defensively.”).

The cultivating phase of CBASP@YoungAge is based on a modular course and can be personalized by the therapists in the modules’ order and implementation. It focuses on the Kiesler-Circle-Family-Training and situation analysis, as well as on additional symptoms requiring treatment in additional sessions. The Kiesler-Circle-Family-Training aims to change learned interpersonal behaviors. Past experiences are actively targeted with the patient and relevant others. The most important aim of the Kiesler-Circle-Family-Training setup is the enhancement of flexibility in interpersonal behavior with different family members/significant others. Participants are encouraged to distinguish between different positions in the Kiesler-Circle and are further trained in the perception and reflection of their behavior, evaluation of behavior effects and behavior adaptations to successfully master difficult interactions. During this module, caregivers participate in every session. Situation analysis is applied to enhance the patient’s self-efficacy and ability to act in difficult situations with other people. Patients are trained to notice that they can be in charge and actually change difficult situations. They learn how to shape situations differently to achieve new, changed outcomes for themselves.

The consolidation phase focuses on relapse prevention, reviews the therapy, and checks whether previously set goals have been achieved.

CBASP@YoungAge covers 20 to 30 sessions. Frequency is at least once a week and up to three times a week. It is up to the therapist to decide which modules to use and when based on the individual patient and her/his caregivers. After treatment completion, booster sessions can take place.

#### Treatment-as-usual control group

The TAU control group is implemented in two cooperating outpatient clinics. The responsible therapists are also psychotherapists in training who are under regular case supervision.

Treatment is based on psychotherapy guidelines [63] and current evidence-based practices for the treatment of depression in childhood and adolescence. No further restrictions with respect to duration or length are made. Family involvement is based on the current psychotherapy accounting rules of the German health care system (every 4th session).

#### Therapists training and adherence to the study protocol

All therapists are graduates in psychology or pedagogics and participate in a postgraduate training as child and adolescent psychotherapists in a university integrated outpatient clinic. As part of this training, they attend theoretical seminars and are supported by regular, high-frequency case supervision every fourth session. Consequently, all therapies in the study are implemented under this regular case supervision. In the intervention group (CBASP@YoungAge), the therapists participate in an additionally CBASP@YoungAge Workshop (ELB, ND) before initiating therapy. The treatment process is accompanied by regular additional CBASP@YoungAge case supervision. Supervision takes place every four weeks and is done by a licensed and CBASP-certified therapist (ELB). The therapists can discuss their cases during the supervision, review the process, and ask questions about the study procedure.

To ensure adherence to the protocol, we created a standardized checklist for data collection for each child/adolescent participating in the study. The study team review the checklists and remind the therapists either by text message (e-mail) or phone calls once a month to complete assessments with their patients in case of missing data. Every module closes with an adherence check that the study therapists complete at the end of each module. As part of the process evaluation, study therapists fill out a documentation form and report on the format, session length, participating persons, the module conducted, and important additional information after each session. The study team reviews all completed forms once a month and contacts the treating therapist in case of difficulties or non-adherence.

For organizational matters, and beyond the therapy implementation, a written study plan is made available to all study therapists. This document explains all study procedures (time points, documents) and the study coordinator (ND) clarifies questions and reviews all documents submitted by the therapists regularly.

### Outcomes

Assessments are at pre-treatment (T1), post-treatment (T2) and follow-up at 6 months (T3) (see Fig. 1 for overview). Assessments are face-to-face (clinical interview) or paper-pencil questionnaires and filled out by index patients and/or their caregivers/significant others.

Primary outcomes are, first, the feasibility of the CBASP@YoungAge treatment program. This includes both practicability in the outpatient setting for therapists and suitability for patients and family members, primarily measured by continuous process evaluation (therapists, patients, parents), therapist adherence, patient dropout rate, and recruitment rate during the study and treatment. Second, change in depressive symptomatology between pre- and post-assessment is a primary endpoint, with change measured by the clinical interview (child DIPS, parent or child version) and the Mood and Feelings Questionnaire (MFQ).

Secondary outcomes are the interpersonal behavior between caregiver and children (Impact-Message-Inventory for children and adolescents, IMI@YA), the global health of both caregivers and participating children (KIDSCREEN; Positive mental health questionnaire, PMH), parents' mental health (Brief-Symptom-Inventory, BSI), and parenting quality (Parenting Questionnaire, EFB).

#### Demographic data

For patients, demographics include information on sex, age, place of birth, spoken languages, siblings, school and social factors like friendships, and sports club affiliation. For caregivers/significant others we assess sex, age, education level, and marital status as well as socio economic status (SES). We also screen for any abnormalities in early development or during pregnancy.

#### Diagnostic Interview for Mental Disorders for Children and Adolescents (Kinder-DIPS)

The Kinder-DIPS [52] is a structured interview for the assessment of mental disorders and behavioral problems in children and adolescents that can be completed with the child/adolescent or their caregiver. The diagnostician decides with whom the interview will be conducted. In most cases, children older than 12 years of age can participate the interview themselves. If there is no clear result, the interview can also be conducted with both parties, i.e., caregiver and child. The Kinder-DIPS provides a diagnostic classification according to DSM-5 [51] or ICD-10.

The Kinder-DIPS contains both open and closed questions. Although both the wording and order of the questions are clearly specified, it is possible for the interviewer to ask clarifying follow-up questions. The interrater reliability of the parent and child versions of the Kinder-DIPS is highly satisfactory with respect to major diagnostic categories, and for ruling out mental disorders [64].

#### Impact Message Inventory (IMI) for children and adolescents (IMI@YA)

The IMI@YA is an adaptation of the German version of the IMI [65] for children and adolescents (Dippel N, Brakemeier E-L, Christiansen H: Capturing impact messages in parent - child interactions: Adaption and Validation of the Impact-Message-Inventory, in preparation). It is an interpersonal questionnaire designed to represent the various dimensions of the Kiesler-Circle that is administered to assess interpersonal characteristics by others [66]. This instrument assesses the interpersonal behavior between caregiver and child, that is, the children provide their perception of their parents’ interaction behavior, and vice versa.

The IMI@YA contains 64 items with eight items per scale to be answered on a 4-point Likert Scale from “not at all” (0) to “very much so” (4). The eight scales represent the octants of the Kiesler-Circle read counterclockwise from the top of the circle are called dominant, hostile dominant, hostile, hostile submissive, submissive, friendly submissive, friendly, and friendly dominant. Cronbach’s alpha ranges between .45 (friendly submissive) and .85 (friendly) in a non-clinical sample (Dippel N, Brakemeier E-L, Christiansen H: Capturing impact messages in parent - child interactions: Adaption and Validation of the Impact-Message-Inventory, in preparation).

#### Mood and Feelings Questionnaire (MFQ)

The MFQ detects clinically relevant symptoms of depression in children and adolescents. For the current study, we use the German version of the MFQ that measures depressive symptoms on a 3-point Likert scale with 0 = “not true”, 1 = “sometimes” and 2 = “true” (Universität Ulm, 2015 based on [67]). The MFQ demonstrates excellent reliability scores (US sample: self: α = .89, caregiver α = .91; German sample: self: α = .93, caregiver: α = .90) [68].

#### KIDSCREEN

The KIDSCREEN [69] assesses children’s and adolescents’ subjective health and well-being. The KIDSCREEN is a self-report measure for healthy and chronically ill children and adolescents aged 8 to 18 years. It is available in different versions. We use the 27-item version in for pre-, post-, and follow-up assessments, and the 10-item short version for therapy process evaluation every 5th session. The KIDSCREEN-27 is based on the 52-item full version and provides detailed profile information on five Health-Related Quality of Life (HRQoL) dimensions. The KIDSCREEN-10 Index provides a global HRQoL score for monitoring and screening purposes and takes 5 min to fill out.

The KIDSCREEN instruments are available in child, adolescent, and parent versions, and have been translated and adapted for use in several languages. A total score can be calculated, and T-values and percentages are available for each country stratified by age and gender. Cronbach’s alpha ranges between .79 (physical wellbeing) to .84 (psychological wellbeing); Cronbach’s alpha is .82 for the ten-item version [70].

#### Positive Mental Health Scale (PMH)

The Positive Mental Health Scale (PMH-scale [71]) measures positive mental health with a brief, unidimensional, and person-centered questionnaire. The scale consists of nine items rated on a Likert scale from 1 = “not true” to 4 = “true”. The internal consistency is about .93 for all groups together and .82 in the retest sample. The PMH-scale’s internal consistency is high and similar across different samples [71]. This instrument is completed by the caregivers.

#### Brief symptom inventory (BSI)

The Brief Symptom Inventory [72] consist of 53 items, and is a questionnaire for documenting subjective impairment caused by physical and especially psychological symptoms. Based on the Symptom Checklist SCL-90-R, the items assign to nine scales (somatization, obsessiveness, insecurity in social contact, depressiveness, anxiety, aggressiveness/hostility, phobic anxiety, paranoid thinking, psychoticism) and enable three indices of global distress to be calculated. The internal consistencies of the nine scales range from .70 (Psychoticism) to .87 (depressiveness). Gender- and age-specific norm values are available. This instrument is also completed by the caregivers.

#### Erziehungsfragebogen (EFB)

The Parenting Questionnaire (EFB) [73] is the German version of the parenting scale [74]. The EFB is a self-assessment scale of parenting behavior in situations when the child is to be disciplined. Its three scales (overreaction, laxness, and verbosity) and a total score can be analyzed. The internal consistencies of the overreacting and laxness scales and of the total score are acceptable to good (.59–.80). The verbosity scale’s values were low [73].

#### Therapy process evaluation

An evaluation is implemented for continuous therapy support by relying on the MRC guidance [75] on how to conduct process evaluations of interventions. This consists of short questionnaires to be completed by everyone involved after each therapy session, and of short questionnaires (KIDSCREEN-10, PMH) after every fifth therapy session. As the study’s target population are youth patients with depression and stressful family relationships, process evaluation is as short and practicable as possible. We therefore limit ourselves to documenting the change in symptoms compared to the beginning of therapy (subjective rating of 1 = “worse” to 10 = “better”), the satisfaction with the actual therapy session (subjective rating 1 = “dissatisfied” to 10 = “satisfied”) and ask for qualitative descriptions of other comments. These open-ended questions are not intended to be a substitute for careful qualitative analysis, but allow us to capture unanticipated therapy events from the participants' perspective not yet included in the protocol. The aim of this first step in the process evaluation is to check the intervention’s feasibility and establish the course of symptoms over time as well as the participants’ general health-related well-being.

In the second step, we focus specifically on the structure of each therapy session by asking therapists how they conduct each session. For that purpose, they fill out a session log sheet after each session. Here they indicate how long the session lasted, who participated, which techniques they applied, and whether the session was conducted online or face-to-face. This will help us understand the feasibility and implementation of each module and session, and optimize the program after this study.

Furthermore, we focus specifically on the intervention’s structure. Have the therapists implemented it correctly? The therapists fill out a session protocol sheet after each session for just this reason. Here they indicate the frequency and duration of the sessions, who participated, and whether the appointments were implemented online or live. This helps us better understand the feasibility and implementation of each module and to identify potential structural differences related to specific modules.

### Adverse events and risk management

During the general psychotherapy training and especially during the CBASP@YoungAge Workshop, study therapists are prepared for unexpected and adverse events. In case of such an event during the CBASP@YoungAge intervention, the study coordination (ND) is informed immediately, and (serious) adverse events are reported to the entire study team (HC, ELB). Detailed information on the definition of (serious) adverse events and detailed documentation procedure within the study can be found in Additional file 3. The initiation of pharmacological treatment during study therapy in the intervention group is also reported to the study coordinator (ND). The dose, duration, and type of medication are documented. In the TAU control group, medication can be part of the standard treatment course.

Individuals with depression carry a higher risk of self-harm and suicide than the general population. Thus, all trial participants are monitored by their responsible outpatient clinic. In the case of patients with an increased risk of acute suicidal tendencies, regular explorations are carried out by the appropriately trained study therapists. In case of uncertainty, an experienced therapist on duty is called in. This represents the standard procedure of the outpatient clinics and thus also of the study. It includes regular consultation with the outpatient clinic management and responsible supervisors of the study therapists. Participants will be able to contact the study coordinator (ND) during the trial period until the end of the follow up assessment.

### Statistical analysis

A preliminary analysis of the results after post-assessment is planned. A full analysis will follow the completion of all follow-up assessments. Data will be analyzed based on the Intention-to-treat (ITT) sample and will help inform power calculations for a future CBASP@YoungAge randomized control trial.

Central aspects with regard to feasibility in the analysis are attrition and reasons for drop-out of patients or parents. These aspects will be recorded and analyzed. Recruitment rate is measured by reporting the number and reasons of patients screened for the intervention group who decided against study participation. We also capture the proportion of patients who participate in each arm at pre-, post- and 6-month follow-up assessment. Furthermore, we access the proportion of patients meeting criteria for response and remission based on the Kinder-DIPS at the different time points. With regard to attrition, therapists will assess the number of sessions of the modules, and the session frequency in general and related to the different modules. In addition, we will record each participant’s individual course of therapy in self-reports, and reports from others, as well as any unexpected events in the course of therapy. For participants who withdraw from the study, the researcher will follow-up with either the therapists or participants to document their reason(s) for study withdrawal. We will assess rates and types of missing data, and the proportion of drop-outs at different stages throughout the trial. We will also assess whether any of the measures display floor and/or ceiling effects. Patient adherence to treatment will be indexed by the mean (SD) number of therapy sessions offered and attended, and by calculating the proportion of participants who undergo a minimum adequate dose (eight or more sessions) in each arm. Adherence of protocol by therapists is analyzed descriptively by the proportion of complete adherence checks and the monitoring of individual case and data management in the study routine. Data completeness is reported as proportions of missing values. Due to sample size and this study’s pilot character, the feasibility analysis is mainly based on the intervention group’s process evaluation. First-interim analyses of results are only possible with sufficient post-measurements. Missing values will be integrated in an iterative imputation method based on a random forest model.

We intend to carry out analyses with regard to effectiveness. Summary statistics will be used as appropriate to describe and compare data for all participants. ANCOVA models will be used to test the pre- and post-differences in the intervention group and the difference between intervention and control group after the intervention and at follow up. Within or between-group effect sizes (Cohen’s *d*) will be calculated and interpreted according to standard conventions (< .50 = small; .50–.80 = medium; > .80 = large [76]).

### Trial management and governance

Clinical notes, measures, and therapy recordings are stored in a locked filing cabinet in a locked office at Philipps-University Marburg. Consent forms are stored separately from data. Data is entered into an SPSS spreadsheet maintained by the trial manager (ND), stored securely on Philipps University Marburg server. Only individuals authorized by the principal investigator have access to the database. We design this first pilot study for conciseness and minimizing risk. Therefore, no formal data monitoring committee has been organized. The completeness and correctness of the data storage will be checked and ensured every two weeks by the study coordinator (ND). The results will be reported to the leading investigators (HC and ELB).

Published material will not contain patient-identifiable information. The datasets generated and/or analyzed will be stored in a repository at Philipps-University Marburg. Anonymized data may be accessed and analyzed by members of the project with researchers collaborating with team members on the analysis of these data. Therefore, external researchers wishing to access the data for future projects or analyses must do so via request to the principal investigator (ND).

## Discussion

The first aim of this study is to apply CBASP, which is well-developed and already evaluated for adults, for the first time in a version modified specifically for children and adolescents with depressive disorders. We aim to focus on integrating caregivers and interpersonal relationships to treat depressive disorders in children and adolescents in an age-appropriate manner relying on the CBASP treatment approach. Attachment and the relationship between parents and children are areas of high relevance in the treatment of depressive disorders and should be considered especially in the context of dysfunctional interaction behaviors or even childhood maltreatment. For this reason, CBASP@YoungAge focuses on involving the actual caregivers as well as on the processing of earlier relationship experiences and their impact on the disorder (“significant others”). We expect that this treatment will result in a stronger alleviation of the young person’s depressive symptoms, and in improved interpersonal behavior of children and caregivers, thus leading to a long-term improvement in the patients’ symptomatology. Since there are so few therapeutic approaches that effectively treat depression in this age group [21], and the current treatment programs reveal such low effect sizes [20], we see an urgent need to investigate a therapy program that addresses both the present symptoms of young people and the interpersonal problems with their actual caregivers. CBASP@YoungAge offers this opportunity and is thus a promising approach to treat depression in children and adolescents.

In addition to the results in terms of clinical relevance, the practicality and feasibility of the trial are also important. Those results will allow us to evaluate the feasibility and effectiveness of this treatment program and will provide valuable information on whether this modular and interaction-focused treatment can actually be implemented in other outpatient treatment settings at different locations. In particular, its feasibility from the perspective of the patients and their relatives, as well as from the therapists will be examined. Any obstacles can be removed and necessary changes can be made according to the results. Furthermore, building on this non-randomized pilot study’s results, we will be able to plan a large controlled randomized trial. Whether this is applicable depends on various factors and aspects. Central is whether the CBASP@YoungAge treatment does not cause harm to patients or relatives. Furthermore, it is important to know whether recruitment is possible in order to implement a larger study and if the therapy can be carried out and terminated by therapists and patients with a reasonable amount of effort. Aspects that are also considered in the evaluation of feasibility are cost-effectiveness, duration of therapies and study as well as effectiveness and feasibility of data management.

However, the results we expect from this pilot study are also hampered by certain limitations. Firstly, it is based on a quasi-experimental design. Since all participants in the intervention group are treated in the Marburg outpatient clinic and all TAU participants are treated in either the outpatient clinic in Bochum or Landau, no randomization is possible, which also means that study-site effects cannot be excluded. Secondly, due to the Corona pandemic, we could not start collecting data at all three outpatient clinics at the same time. That makes seasonal side effects possible between the intervention and control groups (also due to the respective pandemic situation). Third, the design is focused on assessing the feasibility of the treatment program. However, this leads to the fact that not all relevant aspects, e.g., the therapeutic alliance, can be captured. Fourth, because of the ongoing pandemic situation, we were unable to define numeric progression criteria. Regarding the various safety regulations, the rules for outpatient psychotherapy were and are frequently changed. Thus, it was not possible to predict exactly how recruitment, duration of therapy, or even data management would proceed. However, we will carefully monitor the different progression criteria to determine whether progression is feasible and to learn how to adapt and improve progression in a large-scale RCT trial.

Finally, the study’s design means that patients and therapists cannot be blinded which could create some bias among therapists and patients. However, we are trying to mitigate possible biases resulting from the lack of blinding by ensuring that the clinical evaluations are not done by us study therapists but rather by independent blinded raters. Against the background of the first investigation of this intervention within the framework of a phase 1/phase 2 clinical trial, these limitations appear in general justifiable.

## Conclusion

This pilot study is the first to explore the feasibility and effectiveness of CBASP adapted for children and adolescents suffering from depression. Considering the current low treatment outcomes [15] as well as the high proportion of interactional problems in the context of depressive disorders [77, 78], this treatment program is highly relevant. It will provide important knowledge on the feasibility and effectiveness of this program, and reveal the potential of including caregivers in psychotherapy. A multi-center randomized controlled study is being planned based on this study’s results, with the long-term aim of improving psychotherapeutic care for young patients with depression while preventing persistent courses of depressive disorders.

### Trial status

This trial was registered on the German Clinical Trials Register, DRKS (identifier DRKS00023281). The trial opened to recruitment on November/2020, and the first patient was included on 09.11.2020. Notably, the COVID-19 pandemic, including security measures, is slowing recruitment and making it difficult to conduct the study. The recruitment in the intervention group is anticipated to be completed in September 2022 and in the control group in December 2022. The preliminary analysis will then be completed in December 2022. The final follow-up should be completed by the end of July 2023. Data analysis and reporting is expected to take another 6 months.

## Supplementary Information

**Additional file 1.** SPIRIT 2013 Checklist.

**Additional file 2.** WHO Trial Registration Data Set.

**Additional file 3.** Management of adverse events.

## Data Availability

Findings will be disseminated to participants, at the local level through a written summary of results and public engagement events and at a national and international level via conference presentations. After the (interims and final) data analysis are completed, the results will be published. Data will be kept for 10 years after it is analyzed and published. Data will be held in a secure environment on servers of the Marburg University research data repository, a research data repository for faculty, students, and staff. All IPD that underlie results will be available upon request.

## Abbreviations

ANCOVA: Analysis of covariance
BSI: Brief-Symptom-Inventory
CBASP: Cognitive Behavioral Analysis System of Psychotherapy
CONSORT: Consolidated Standards of Reporting Trials
DPI: Disciplined Personal Involvement
DSM-5: Diagnostic and Statistical Manual of Mental Disorders
EFB: Parenting Scale (German adaption)
IMI@YA: Impact-Message-Inventory(@YoungAge) for children and adolescents
IQ: Intelligence Quotient
ITT: Intent-to-treat
Kinder-DIPS: Diagnostic interview for mental disorders in childhood and adolescence
KJ-PAM: Child and Adolescent Outpatient Clinic Marburg
MFQ: Mood and Feelings Questionnaire
MRC: Medical Research Council
PMH: Positive-Mental-Health scale
RCT: Randomized controlled trial
SES: Socio-economic status
SPIRIT: Standard protocol items: recommendations for interventional trials
TAU: Treatment as usual
