# Correction: Investigating the feasibility and effectiveness of a modular treatment program for children and adolescents with depression and interpersonal problems: study protocol of a quasi-experimental pilot feasibility trial (CBASP@YoungAge)

**DOI:** 10.1186/s40814-022-01118-9

**Published:** 2022-07-27

**Authors:** N. Dippel, T. In-Albon, S. Schneider, H. Christiansen, E.-L. Brakemeier

**Affiliations:** 1 Philipps-University Marburg, Marburg, Germany; 2 University of Koblenz-Landau, Landau, Germany; 3 Ruhr-University Bochum, Bochum, Germany; 4 University of Greifswald, Greifswald, Germany

**Correction: Pilot Feasibility Stud 8, 145 (2022)**

**https://doi.org/10.1186/s40814-022-01091-3**

Following publication of the original article [1], the authors reported an error on page 8 wherein the details for reference Dippel et al is incomplete. The correct details of the reference is given below.

Dippel N, Zimmermann J, Brakemeier E-L, Christiansen H. Capturing impact messages in parent - child interactions: Adaption and Validation of the Impact-Message-Inventory. In preparation.

The original article [1] has been updated.
