# Supplementary material for: Investigating the feasibility and effectiveness of a modular treatment program for children and adolescents with depression and interpersonal problems: study protocol of a quasi-experimental pilot feasibility trial (CBASP@YoungAge)
Source: Pilot Feasibility Stud. 2022 Jul 11;8:145. doi: 10.1186/s40814-022-01091-3 (PMC9275387; doi:10.1186/s40814-022-01091-3)
Supplement: Supplementary file 2 — Additional file 2. WHO Trial Registration Data Set. [file 40814_2022_1091_MOESM2_ESM.docx]

Additional file 2

| **WHO Trial Registration Data Set** | |
| --- | --- |
| 1. **Primary Registry and Trial Identifying Number** | German register of clinical studies (DRKS), DRKS00023281 |
| 1. **Date of Registration in Primary Registry** | 11-17-2020 |
| 1. **Secondary Identifying Numbers** | Universal Trial Number (UTN): U1111-1259-5590 |
| 1. **Source(s) of Monetary or Material Support** | Institutional budget, no external funding (budget of sponsor/PI) |
| 1. **Primary Sponsor** | Philipps-University of Marburg |
| 1. **Secondary Sponsor(s)** | - |
| 1. **Contact for Public Queries** | Philipps-University of Marburg  Dipl.-Psych. Nele Dippel  Gutenbergstraße 18  35032 Marburg  Germany |
| 1. **Contact for Scientific Queries** | Philipps-University of Marburg  Dipl.-Psych. Nele Dippel  Gutenbergstraße 18  35032 Marburg  Germany |
| 1. **Public Title** | - |
| 1. **Scientific Title** | Investigating the feasibility and effectiveness of a modular treatment program for children and adolescents with depression and interpersonal problems: study protocol of a quasi-experimental pilot feasibility trial (CBASP@YoungAge) |
| 1. **Countries of Recruitment** | Germany |
| 1. **Health Condition(s) or Problem(s) Studied** | Depression in children and adolescents |
| 1. **Intervention(s)** | Arm 1*: CBASP@YoungAge: Outpatient treatment program  Arm 2*: CBT in routine clinical care (standard outpatient psychotherapy treatment according to guidelines for psychotherapy)  *both arms are carried out at German university outpatient clinics |
| 1. **Key Inclusion and Exclusion Criteria** | **Inclusion Criteria**   - Age between 10 and 21 years - The index patient meets the criteria for depression as the primary diagnosis (according to DSM-5) - The index patient and her/his participating caregiver are fluent in German language and writing - The intelligence quotient of the index patient is ≥ 80 (assessment not older than 2 years)   **Exclusion Criteria**   - Already running psychotherapeutic treatments - Severity of the disorder or comorbid disorder requiring an inpatient stay or other treatment prior to study participation. - Acute suicidal ideation of the index patient |
| 1. **Study Type** | Interventional, prospective, non-randomized controlled trial |
| 1. **Date of First Enrollment** | 11-09-2020 |
| 1. **Sample Size** | 44 (Target) |
| 1. **Recruitment Status** | ongoing |
| 1. **Primary Outcome(s)** | Change in severity of depressive symptomatology from pre- to post-treatment, operationalized by the Kinder DIPS (child or parent version) and Mood and Feelings Questionnaire (MFQ) |
| 1. **Key Secondary Outcomes** | 1. Change in interpersonal behavior between participating caregivers and patient from pre- to post-treatment as measured by the IMI-KJ.  2. Change in parenting behavior from pre- to post-treatment time point. Measured with the EFB in the self-report by caregivers.  3. Change in global health index of the index patient after every five sessions during treatment.  4. Change in health status of the participating caregivers after every five sessions during treatment. |
| 1. **Ethics Review** | File number 2020-75k, Marburg - Ethics Committee of the Department of Psychology of the Philipps University Marburg |
| 1. **Completion date** | Anticipated date of last data collection (Follow-up): 2023/06/31 |
| 1. **Summary Results** | Analyses have not been conducted yet. |
| 1. **IPD sharing statement** | All IPD that underlie results will be available upon request. Data will be kept for 10 years after it is analyzed and published. Data will be held in a secure environment on servers of the Marburg University research data repository, a research data repository for faculty, students and staff. |
