# Supplementary material for: Investigating the feasibility and effectiveness of a modular treatment program for children and adolescents with depression and interpersonal problems: study protocol of a quasi-experimental pilot feasibility trial (CBASP@YoungAge)
Source: Pilot Feasibility Stud. 2022 Jul 11;8:145. doi: 10.1186/s40814-022-01091-3 (PMC9275387; doi:10.1186/s40814-022-01091-3)
Supplement: Supplementary file 3 — Additional file 3. Management of adverse events. [file 40814_2022_1091_MOESM3_ESM.docx]

**Additional File 3**

**Management of adverse events**

Adverse events in the study are defined orientated on the CONSORT (CONsolidated Standards of Reporting Trials) 2010 guideline as potentially harmful events that could occur to patients in clinical trials, regardless of whether they are in the intervention or control group. The adverse event does not necessarily have to be related to the study.

1. Adverse Events in the course of therapy can include the following aspects:
   - symptoms deteriorating more than expected
   - unexpected novel symptoms occurring during the therapy course
   - acute suicidal tendencies (plans or actions)
   - unexpected disturbances in the therapeutic relationship
2. Serious adverse events are:
   - death
   - life-threatening situations
   - or unexpected inpatient stays
   - an important medical event

All events that are unanticipated from the therapist's perspective are documented as unexpected events.

**Reporting of Serious Adverse Events and Unanticipated Problems:**

In the case of a potentially (serious) adverse event, the therapists contact the study coordinator (ND) as soon as possible. All unexpected events that occur are recorded by the therapist at their center using a standardized form. Any other procedures are coordinated with the therapist, responsible case supervisor, and with the CBASP@YoungAge supervisor. If we can rule out any connection between the event and CBASP@YoungAge treatment program, the treatment proceed as planned. If such a connection cannot be excluded, the therapy discontinued, and regular treatment takes place. If the treatment is discontinued by the patient (e.g., due to lack of motivation), the patient is documented as a drop-out including the reason. During the trial’s active treatment phase, participants are asked not to participate in any other psychological therapy (reflecting standard practice that only one therapy can be completed at a time).

The management of adverse events during treatment in the TAU condition is consistent with the corresponding usual practices of recruitment centers. This includes that risks such as suicidality are closely monitored by the regular case supervision in the intervention and control condition.
